# Intraventricular Spring Expander Attenuates Cardiac Atrophy of the Failing Heart After Unloading Caused by Heterotopic Heart Transplantation: No Sex-Linked Differences

**DOI:** 10.33549/physiolres.935560

**Published:** 2025-06-01

**Authors:** Dushan Michael KOLESÁR, Iveta MRÁZOVÁ, Petr KUJAL, Martin POKORNÝ, Petra ŠKAROUPKOVÁ, Janusz SADOWSKI, Michal ŠNOREK, Zdeněk ČERMÁK, Karel VOLENEC, Barbara SZEIFFOVÁ BAČOVÁ, Matúš SÝKORA, Luděk ČERVENKA, Ivan NETUKA

**Affiliations:** 1 Department of Cardiovascular Surgery, Institute for Clinical and Experimental Medicine, Prague, Czech Republic; 2 Third Faculty of Medicine, Charles University, Prague, Czech Republic; 3 Center for Experimental Medicine, Institute for Clinical and Experimental Medicine, Prague, Czech Republic; 4 Department of Pathology, Third Faculty of Medicine, Charles University, Prague, Czech Republic; 5 Department of Cardiology, České Budějovice Hospital, České Budějovice, Czech Republic; 6 ELLA-CS, Ltd. Hradec Králové, Czech Republic; 7 Center of Experimental Medicine, Institute for Heart Research, Slovak Academy of Sciences, Bratislava, Slovak Republic

**Keywords:** Heart failure, Cardiac atrophy, Sex differences, Heterotopic heart transplantation, Mechanical heart unloading

## Abstract

Cardiac atrophy is the most common complication of prolonged application of the left ventricle (LV) assist device (LVAD) in patients with advanced heart failure (HF), obviously, it is a consequence of LVAD-induced mechanical unloading. Previous studies employing heterotopic heart transplantation (HT_x_) as a model of heart unloading after LVAD implantation discovered sex-linked differences in the course of unloading-induced in the healthy hearts. It remains to be clarified if sex-related differences are present in the failing hearts after heterotopic HT_x_. Therefore, we first compared the course of unloading-induced cardiac atrophy in the failing hearts in intact (without gonadectomy) male and female rats, and in animals after gonadectomy, to explore the influence of sex hormones on this process. Second, we examined if the animal’s sex modifies the effects of increased isovolumic loading of the LV on the course of unloading-induced cardiac atrophy. Heterotopic abdominal heart transplantation (HT_x_) was used as a rat model of heart unloading. HF was induced by volume overload achieved by creation of aorto-caval fistula. Increased isovolumic loading was obtained by implantation of specially designed three-branch spring expander into the LV. The degree of cardiac atrophy was assessed as the whole heart weight (HW) ratio of the heterotopically transplanted to the native control heart. We found that decreases in HW after HT_x_ were similar in intact male and female rats, similarly in intact and gonadectomized animals. Implantation of the expander significantly and comparably reduced decreases in HW in male and in female rats. We conclude that there are no sex-linked differences in the development of unloading-induced cardiac atrophy in the failing hearts. Our results also show that enhanced isovolumic heart loading obtained using the spring expander attenuates the development of unloading-induced cardiac atrophy in the failing hearts; the degree of attenuation is similar in both sexes.

## Introduction

Along with the paucity of donor hearts, the actual eligibility for heart transplantation (HT_x_) remains a prominent barrier for HT_x_ application for the treatment of patients with end-stage heart failure (HF), even though HT_x_ is still the best therapeutic approach for such patients. Implantation of a durable left ventricular assist device (LVAD), which was originally introduced as a “bridge to HT_x_” has become a standard therapeutic approach in patients with end-stage HF [1–7]. After implantation, LVAD provides a favorable hemodynamic environment for the left ventricle (LV) as it offers rest and alleviation of pressure. It has been reported that under such conditions complex adverse molecular, cellular and structural changes of the LV (characteristic for progression of HF), develop in a process termed “LV remodeling”. This was believed to be irreversible [8–11], yet it can be reversed in a process of “LV reverse remodeling” [12–14]. It has been claimed that the reversal leads to functional recovery, resulting finally in the state where the LVAD becomes unnecessary and can be explanted. Eventually, a new indication for the LVAD use in patients in end-stage HF emerged and was named “bridge to recovery” [1–7,15–21]. However, a recent comprehensive investigation demonstrated that LV function recovery in patients with an indication for “bridge to recovery” is rare [22,23]. Apparently, for unclear reasons the biological signs of LV reverse remodeling are not readily translated into clinical improvement of LV function [12–16,18,24–33]. The reasons for this divergence are definitely multifactorial but it is important to emphasize that there is growing awareness that the long-term LVAD support has also detrimental effects. The widely recognized harmful effect of prolonged LVAD use is the development of cardiac atrophy, a consequence of LVAD-induced mechanical unloading. It has been suggested that a pronounced degree of cardiomyocyte atrophy prevails over the beneficial effects on the biological characteristics; apparently, the latter are not translated into the LV functional improvement [24–29,34,35]. Based on various attempts to minimize unloading-induced cardiac atrophy which were mostly ineffective [27,35–42], it has been recognized that the prerequisite for finding successful treatment approaches minimizing unloading-induced cardiac atrophy is detailed knowledge about the physiology of this process; there is no doubt that our understanding is here incomplete.

To address this problem the model of heterotopic rat HT_x_ was developed and many scientific groups, including our own, have performed studies employing this model and, indeed, ample relevant information was provided [24,27–29,36–41,43]. Until recently, all these studies exhibited a critically important limitation in that they were performed solely in males, while it is known that there are important sex-related differences in the pathophysiology of HF [44–47] and also in the use and outcomes of LVAD [48–51]. Therefore, one should anticipate the existence of sex-linked differences in the process of unloading-induced cardiac atrophy. Our two latest studies addressed the issue of such differences in the course of unloading-induced cardiac atrophy after HT_x_. We found that the unloading-induced cardiac atrophy was substantially less pronounced in female than in male rats [52,53]. Based on the results of those two comprehensive studies, we concluded that the process of unloading-induced cardiac atrophy exhibits important sex-linked differences, and the attenuation of this process in female rats cannot be simply ascribed to the protective effects of estradiol or the absence of deleterious actions of testosterone [52,53]. However, before making ultimate general conclusion about sex-linked differences in the process of unloading-induced cardiac atrophy, another limitation of our recent studies must be addressed. It is important to recognize that our recent studies were performed with unloaded healthy (i.e. non-failing) hearts. Therefore, it is unclear if these sex-linked differences in the development of unloading-induced cardiac atrophy are present in the hearts from animals with advanced stage of HF (i.e. failing hearts), and if this is the case, to what extent. The said limitation is even more important in the light our own previous findings showing that the natural course of unloading-induced cardiac atrophy differs between healthy and failing hearts [40]. Moreover, we found that implantation of the stainless-steel spring expander into the LV (it provides sufficient isovolumic loading without impairing LV ejection function) attenuated the process of unloading-induced cardiac atrophy after heterotopic HT_x_ in the failing [54] but not in the healthy hearts [41]. This further underscores the need for performing studies not only in the healthy but also in the failing hearts.

All things considered, our ***first aim*** was to evaluate if sex-related differences are present in the course of cardiac atrophy after heterotopic HT_x_ in the failing hearts and, if so, what is their nature.

The ***second aim*** was to examine if sex-related differences are present in the course of unloading-induced cardiac atrophy in the failing hearts with implanted LV spring expander. In other words, if sex-linked differences modify the effects of increased isovolumic loading of the LV on the course of cardiac atrophy after HT_x_.

## Methods

### Ethical approval

The studies were performed in agreement with the guidelines and practices established by the Animal Care and Use Committee of the Institute for Clinical and Experimental Medicine, Prague, which accord with the European Convention on Animal Protection and Guidelines on Research Animal Use and were approved by this committee and subsequently by the Ministry of Health of the Czech Republic (the decision number for this project is 18680/2020-4/OVZ).

### Animals, HT_x_ and HF models, and gonadectomy technique

Adult male and female Lewis rats (Charles River Laboratories, Velaz, Prague, Czech Republic), 8 weeks of initial age, were used. The classical heterotopic HT_x_, originally described by Ono and Lindsey [55] and employed and validated by many investigators [24,28,29,35–39,42,43,53] was used as the model to simulate the effect of full mechanical unloading of the heart; its modification was established in our laboratory and has been routinely employed [40,41,52–54]. HF was induced by volume overload induced by aorto-caval fistula (ACF) created using needle technique as originally described by Garcia and Diebold [56] and then employed and validated by many investigators including our own group [57–64]. Eight weeks after ACF creation the animals were used as heart donors. Earlier studies, including ours, demonstrated that at that time ACF animals are in the stage of advanced HF and if untreated soon progress toward decompensated hypertrophy and HF [57–64].

Gonadectomy or sham-operation was performed under combined anesthesia with intraperitoneal ketamine/midazolam mixture (Calypsol, Gedeon Richter, Hungary, 160 mg/kg of body weight, and Dormicum, Roche, France 160 mg/kg of body weight); this was done 28 days before heterotopic HT_x_. The details of the operation were as described in our previous studies [52,53]. Briefly, in female rats, the peritoneal cavity was opened and the ovaries and uterus were removed, thereafter, the peritoneal cavity was cleaned and the muscle wall and skin were sutured. In male rats, orchiectomy was performed: the ductus deferens was isolated and ligated and then each testicle was removed *via* midline incision on the scrotum. Butorphanol (Torbugesic, Fort Dodge Animal Health, Fort Dodge, KS, USA), at the dose of 2 mg/kg of body weight, given every 12 h, was administered subcutaneously for 48-hour postoperative analgesia. In our recent comprehensive study, the effectiveness of gonadectomy was validated in the model of heterotopic HT_x_ [53] and therefore the same protocol of gonadectomy was applied in the present study.

#### Detailed experimental design for evaluation of unloading-induced cardiac atrophy in failing hearts

##### Series 1: The course of cardiac atrophy after heterotopic HT_x_ in failing hearts: sex-differences and effects of castration

**Fig. 1:**
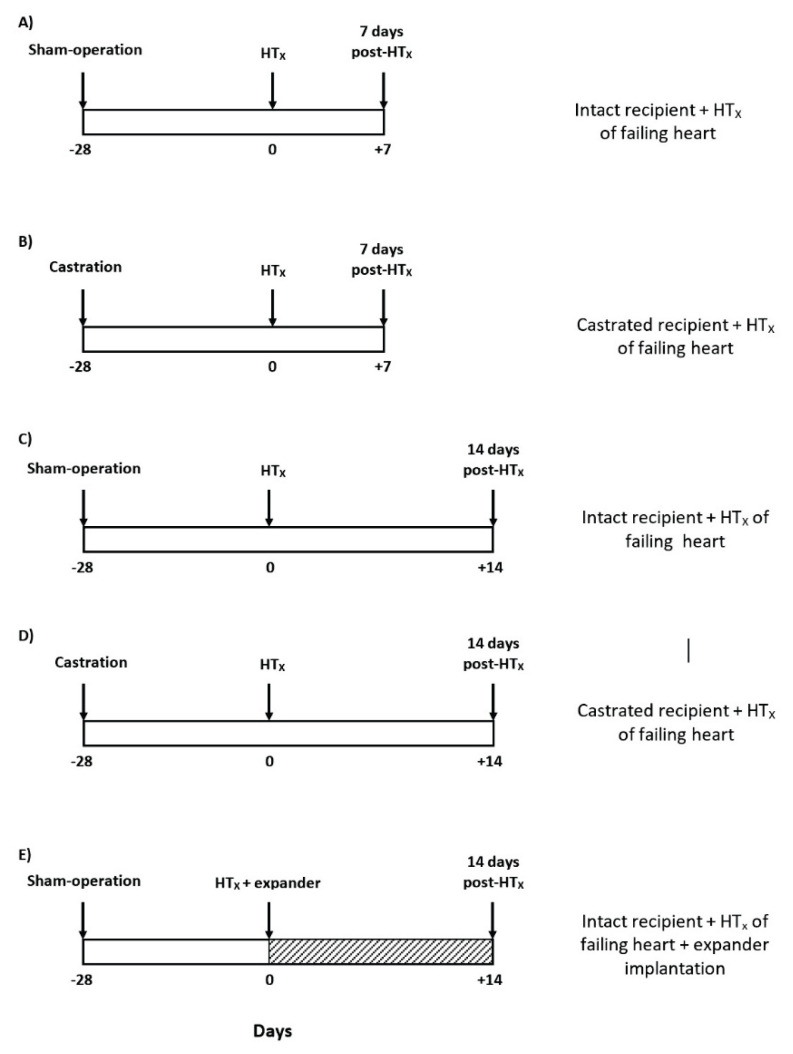
An outline of the set of experiments evaluating the course of cardiac atrophy after heterotopic heart transplantation (HT_x_) to Lewis rat recipients.

**Fig. 2:**
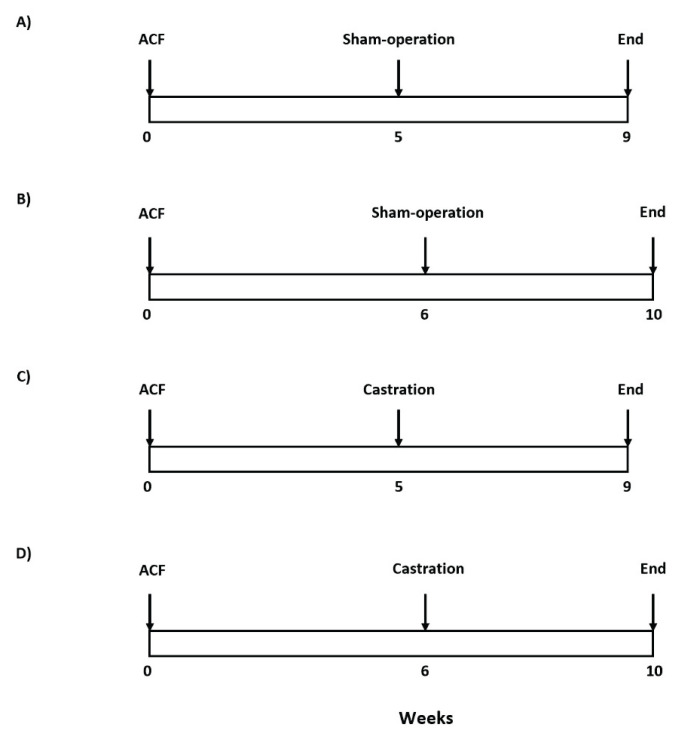
An outline of the set of experiments for control groups of animals after creation of aorto-caval fistula (ACF).

The experimental design used in this series is outlined in Figure 1A–D. On day labeled −28 (i.e. 28 days before heterotopic HT_x_) either sham-operation or castration were performed, and on day labeled 0 the own HT_x_ was performed. We and others [24,28,36,38–41,54] have demonstrated that the unloading-induced cardiac atrophy develops within the first 14 days after HT_x_ when a dramatic loss of myocardial mass is seen. The following 28 days is a steady-state period with no further loss of cardiac mass, suggesting stabilization of unloading-induced cardiac atrophy. In the present study similarly as in our two earlier studies [52,53] the degree of cardiac atrophy was determined 7 days and 14 days after HT_x_ (Fig. 1A–D). Both for donors and recipients the degree of atrophy was assessed from the weight of the total heart and of its individual structural components [LV + septum, and right ventricle (RV)]. Explicitly, the index of cardiac atrophy was calculated as the ratio of the weight of the heterotopically transplanted to the control heart. The degree of cardiac atrophy was expressed as the percent decrease in the whole heart weight (HW), LV weight (LVW), and RV weight (RVW) of the hearts after HT_x_ compared to the control. Unfortunately, HW of the donor’s heart before and after HT_x_ cannot be used for evaluation of the degree of cardiac atrophy, because the heart is immediately placed in cold cardioplegia solution, which precludes precise determination of HW. As control heart served heart from animals with ACF-induced HF, examined 9 and 10 weeks after creation of ACF and again either after sham-operation or castration 28 days before (experimental groups #9 to #16 in the list below and the protocol for obtaining control heart values is outlined in Fig. 2). This was done so because only healthy Lewis rats can be used as recipients: the animals 9 to 10 weeks after creation of ACF develop decompensated HF [57–64] and would not survive the surgical procedure, as tested in preliminary experiments in our previous study which evaluated the course of unloading-induced cardiac atrophy in the failing heart [54]. Control hearts from two different time points were used, i.e. 9 and 10 weeks after ACF creation, because we assumed that in Lewis rats there is still a progressive rise in the development of cardiac hypertrophy after ACF creation. Since these native heart values served as basal values (100 %) for evaluation of the process of cardiac atrophy after HT_x_ it was necessary to use appropriate time control hearts, otherwise the outcomes of analyses would be altered. The following groups were examined (n=9 in each):

1. Sham-operated male Lewis rats (recipient) + HT_x_ of failing male donor’s heart (7 days after HT_x_),
2. Sham-operated male Lewis rats + HT_x_ of failing male donor’s heart (14 days),
3. Castrated male Lewis rats + HT_x_ of failing male donor’s heart (7 days),
4. Castrated male Lewis rats + HT_x_ of failing male donor’s heart (14 days),
5. Sham-operated female Lewis rats + HT_x_ of failing female donor’s heart (7 days),
6. Sham-operated female Lewis rats + HT_x_ of failing female donor’s heart (14 days),
7. Castrated female Lewis rats + HT_x_ of failing female donor’s heart (7 days),
8. Castrated female Lewis rats + HT_x_ of failing female donor’s heart (14 days),
9. ACF male Lewis rats 9 weeks after creation of ACF [control group for intact (recipient) + HT_x_ of failing donor’s heart – 7 days after HT_x_],
10. ACF male Lewis rats 10 weeks after creation of ACF [control group for intact (recipient) + HT_x_ of failing donor’s heart – 14 days after HT_x_],
11. Castrated ACF male rats 9 weeks after creation of ACF [control group for castrated (recipient) + HT_x_ of failing donor’s heart – 7 days after HT_x_],
12. Castrated ACF male rats 10 weeks after creation of ACF [control group for castrated (recipient) + HT_x_ of failing donor’s heart – 14 days after HT_x_],
13. ACF female Lewis rats 9 weeks after creation of ACF [control group for intact (recipient) + HT_x_ of failing donor’s heart – 7 days after HT_x_],
14. ACF female Lewis rats 10 weeks after creation of ACF [control group for intact (recipient) + HT_x_ of failing donor’s heart – 14 days after HT_x_],
15. Castrated ACF female rats 9 weeks after creation of ACF [control group for castrated (recipient) + HT_x_ of failing donor’s heart – 7 days after HT_x_],
16. Castrated ACF female rats 10 weeks after creation of ACF [control group for castrated (recipient) + HT_x_ of failing donor’s heart – 7 days after HT_x_],

at the end of the experiment, the hearts were excised, blood was removed from the chambers by gentle compression, and the hearts’ wet weight was determined.

##### Series 2: Effects of enhanced isovolumic loading induced by implantation of the spring expander into LV of the transplanted heart on the cardiac atrophy after heterotopic HT_x_ in failing hearts: sex-differences

The experimental design used in this series is outlined in Figure 1E. HT_x_ of failing hearts was performed and implantation into the LV of the stainless steel three-branch expander (briefly: “expander”) was performed through LV apex incision. Our previous studies employing echocardiography [41,54] demonstrated that expander implantation did not impair ejection function of the LV and did not cause any mechanical damage of the aortic valve, and there were no signs of serious congestion as compared with transplanted hearts without expander implantation. Therefore, we used this validated expander (branch length of 9 mm), for the failing heart [54]. The composition of the stainless wire (0.17 mm in diameter, 316 LVM, Fort Wayne Metal) used for expander construction, was (%): carbon 0.023, manganese 1.84, silicon 0.37, phosphorus 0.014, sulfur 0.001, chromium 17.57, nickel 14.68, molybdenum 2.79, copper 0.03, nitrogen 0.03 and iron to the balance of 100 %. Elastic and mechanical properties of spring expanders were measured *in vitro* on the miniaturized compression device and analyzed by generation of stress-strain relationship as described by Lossef *et al*. [65]. The following groups were examined in this series:

1. Sham-operated male Lewis rats + HT_x_ of failing male donor’s heart + implantation of expander (14 days) (n=11),
2. Sham-operated female Lewis rats + HT_x_ of failing female donor’s heart + implantation of expander (14 days) (n=10).

In separate experimental groups that are listed below, the hearts were subjected to histological examination of the myocardium as described previously [40,41,52–54]. The histological examination had to be performed in separate groups of animals because perfusion with cardioplegia solution with subsequent fixation in paraformaldehyde solution precludes precise determination of the whole HW weight and the LVW or RVW. Briefly, the rats were anesthetized with a combination of midazolam 5 mg.kg^−1^ (Dormicum, Roche Ltd., Prague, Czech Republic) and ketamine 50 mg.kg^−1^ (Calypsol, Gedeon Richter Ltd., Budapest, Hungary) i.p.. Beating (pulsating) organs i.e. the native heart and the heart after HT_x_ (from the abdomen), were perfused in situ with 20 ml of Thomas cardioplegia solution and subsequently fixed in 4 % paraformaldehyde in phosphate-buffered saline and embedded into Tissue-Tek. The blocks were cut using a cryo-microtome, and cardiomyocyte width was measured in the subendocardium, mid-myocardium, and sub-epicardium of the LV. Cardiomyocyte length was measured only in the mid-myocardium; in each layer, 50 cardiomyocytes were assessed. To avoid underestimation, only the cells in which the nucleus was visible were measured. Since there were no significant differences in the cardiomyocyte width between the layers, the data from the subendocardium, midmyocardium, and subepicardium were pooled as was also practiced by other investigators [66]. Measurements of cardiomyocyte width and length were performed 14 days after heterotopic HT_x_ and appropriate native ACF hearts were used. The following groups were examined in this series:

1. Sham-operated male Lewis rats + HT_x_ of failing male donor’s heart (14 days) (n=9). Native hearts (i.e. from the chest) and HT_x_ hearts (i.e. transplanted to the abdomen) were here examined.
2. Sham-operated male Lewis rats + HT_x_ of failing male donor’s heart + implantation of expander (14 days) (n=10). Native hearts and HT_x_ hearts were here examined.
3. ACF male Lewis rats 10 weeks after creation of ACF (n=9). Native hearts were here examined.
4. ham-operated female Lewis rats + HT_x_ of failing female donor’s heart (14 days) (n=9). Native hearts and HT_x_ hearts were here examined.
5. Sham-operated female Lewis rats + HT_x_ of failing female donor’s heart + implantation of expander (14 days) (n=11). HT_x_ hearts were here examined.
6. ACF female Lewis rats 10 weeks after creation of ACF (n=9). Native hearts were here examined.

### Statistical analyses

All values are expressed as mean ± SEM. Using the Graph-Pad Prism software (Graph Pad Software, San Diego, CA, USA), statistical analysis was done by Wilcoxon’s signed-rank test for unpaired data, or one-way analysis of variance (ANOVA) when appropriate. The values exceeding 95 % probability limits (p<0.05) were considered statistically significant.

## Results

### Series 1: The course of cardiac atrophy after heterotopic HT_x_ in failing hearts: sex-differences and effects of castration

**Fig. 3:**
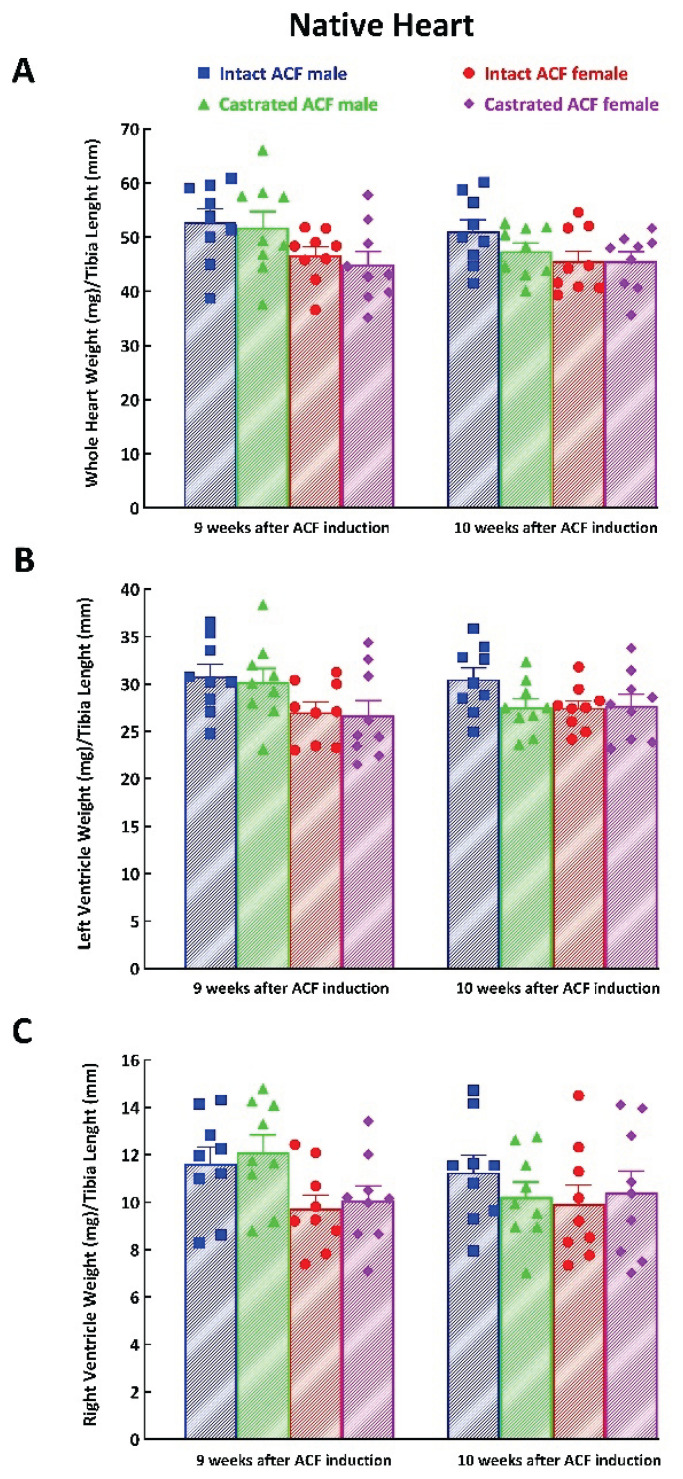
Whole heart weight to tibia length ratio (**A**), left ventricle heart weight to tibia length ratio (**B**) and right ventricle weight to tibia length ration (**C**) in the native heart measured 9 and 10 weeks after creation of aorto-caval fistula (ACF) in intact and castrated male and female Lewis rats.

Tables 1 and 2 collect the absolute values of whole HW, LVW, and RVW of the transplanted hearts obtained 7 and 14 days after HT_x_ in male and female rats and the same parameters for ACF animals. The values for the native heart, either in the chest of the intact or castrated ACF animals at the appropriate time point (i.e. either 9 or 10 weeks after ACF creation) served as basal values (100 %) for evaluation of the process of cardiac atrophy. When the same sex was compared, there were no significant differences between the absolute values in the chest of intact ACF animals versus those in the chest of castrated ACF animals. As expected, the absolute weight of the native hearts in the chest of the intact ACF animals and castrated ACF animals was significantly lower in female ACF rats than in male ACF rats. We normalized the original weight values to the corresponding tibia length and found that there were no differences in the cardiac mass of native hearts between male and female ACF rats (Fig. 3).

**Fig. 4:**
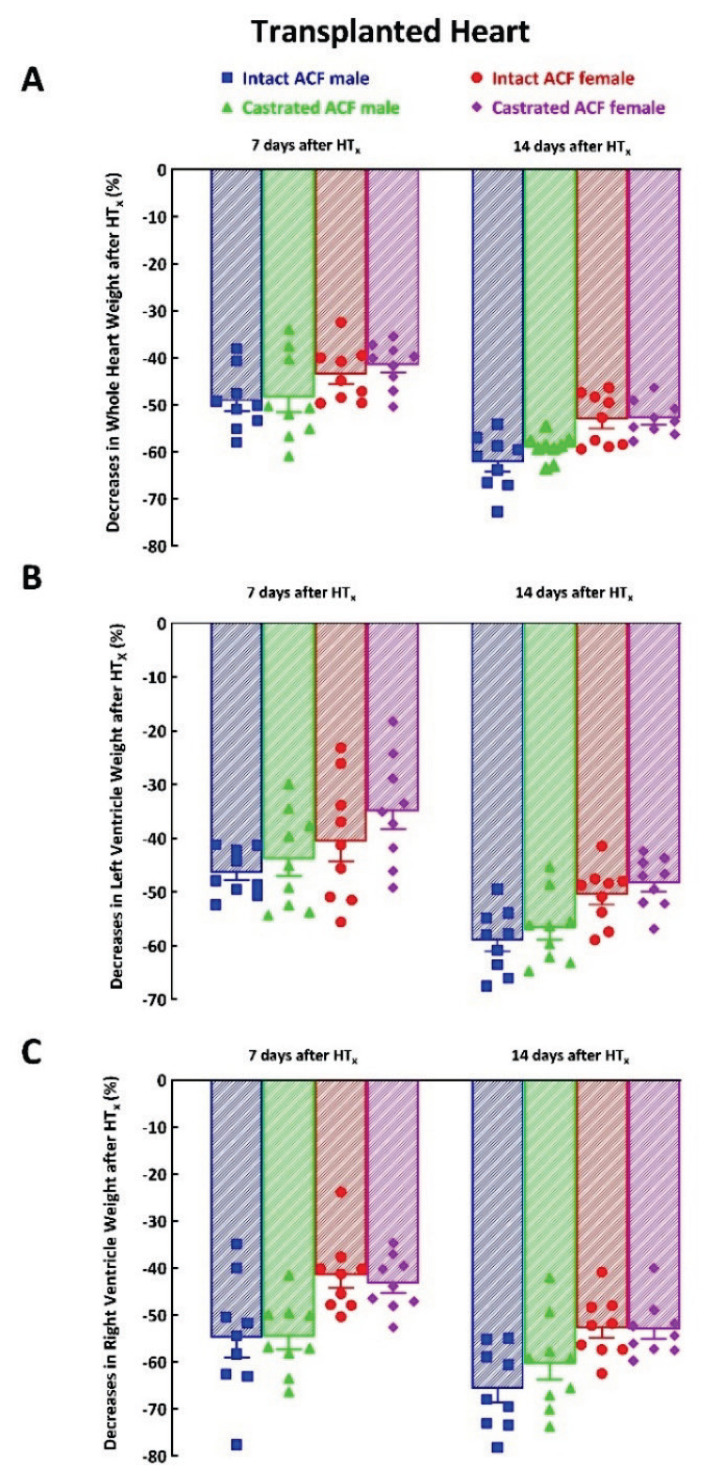
Effects of castration on the course of cardiac atrophy in response to mechanical heart unloading induced by heterotopic heart transplantation (HT_x_) in animals with heart failure elicited by creation of aorto-caval fistula (ACF) in male and female Lewis rats. Data are expressed as percent decreases compared with the native failing heart: (**A**) changes in whole heart weight, (**B**) changes in left ventricle weight, (**C**) changes in right ventricle weight.

As shown in Figure 4A, 7 days’ unloading by HT_x_ in failing hearts resulted in profound, but similar decreases in whole HW in ACF male and ACF female rats under both experimental set-ups (i.e. in intact as well as in castrated animals) (−49±2, −48±3, −44±2 and −42±2 %, P>0.05); the decreases further progressed (see day 14) but still there were no significant differences between experimental groups (−62±2, −59±3, −55±1 and −54±2 %, P>0.05). The dynamics of LVW and RVW decreases was quite similar as that of whole HW (Fig 4B and 4C).

### Series 2: Effects of enhanced isovolumic loading induced by implantation of the spring expander into LV of the transplanted heart on the cardiac atrophy after heterotopic HT_x_ in failing hearts: sex-differences

**Fig. 5:**
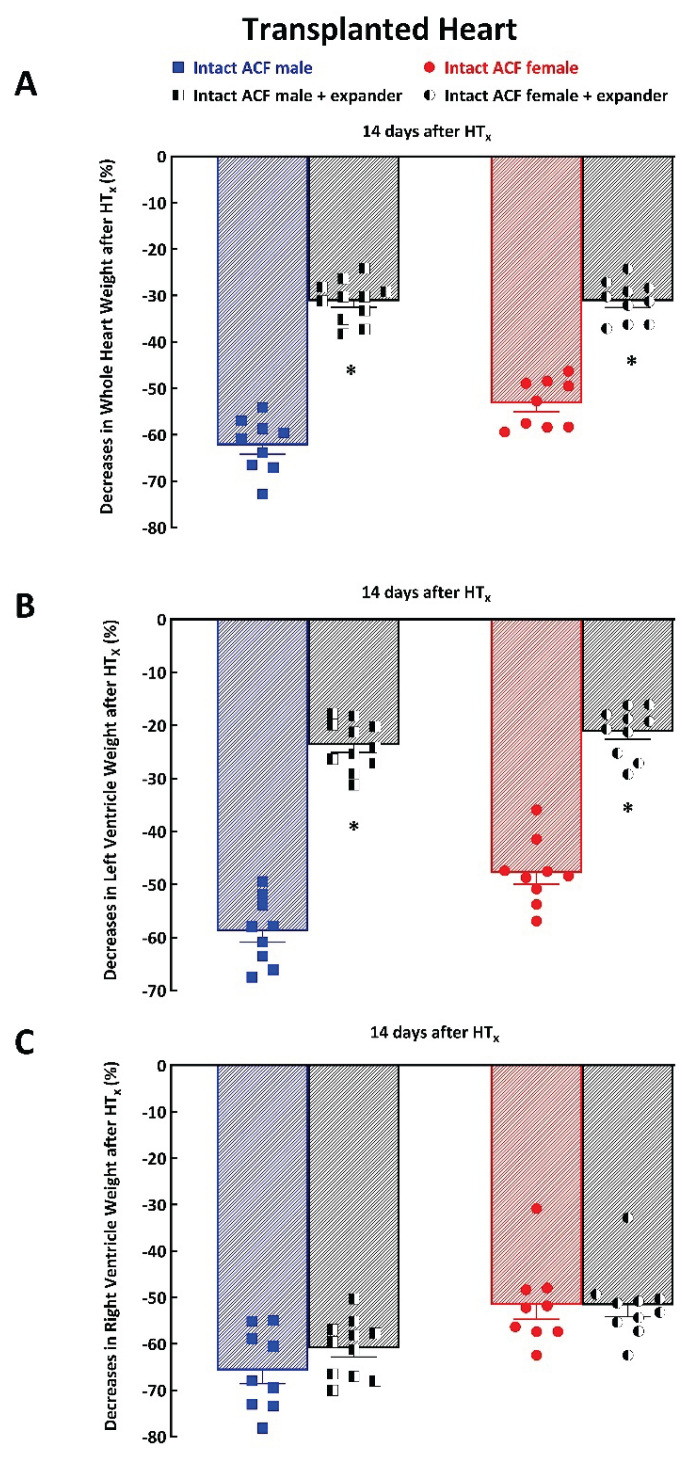
Effect of implantation of the spring expander on the course of cardiac atrophy in response to mechanical heart unloading induced by heterotopic heart transplantation (HT_x_) in animals with heart failure elicited by creation of aorto-caval fistula (ACF) in male and female Lewis rats. Data are expressed as percent decreases compared with the native failing heart: (**A**) changes in whole heart weight, (**B**) changes in left ventricle weight, (**C**) changes in right ventricle weight. * P<0.05 compared with animals without the expander.

**Fig. 6:**
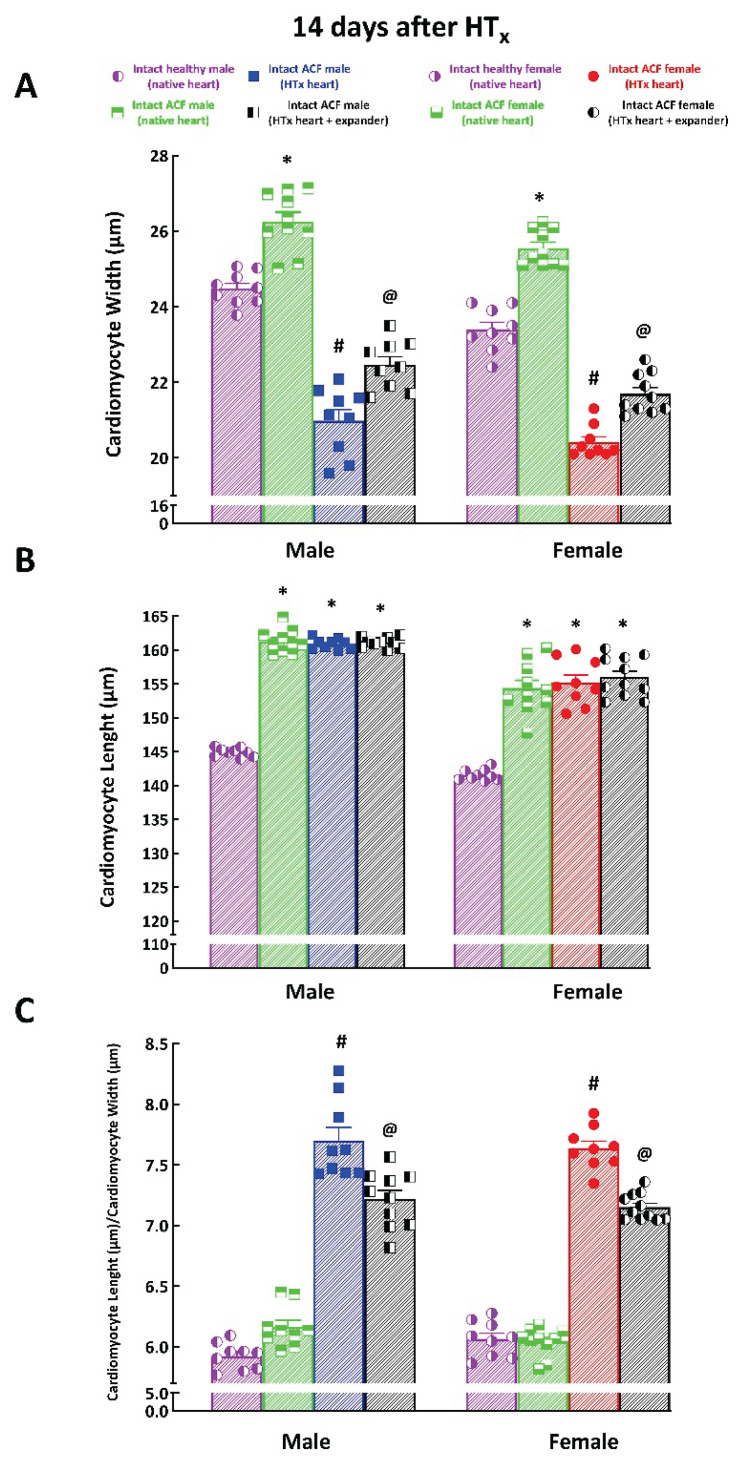
Effect of implantation of the spring expander on the cardiomyocyte size change in the left ventricle in response to mechanical heart unloading induced by heterotopic heart transplantation (HT_x_) in animals with heart failure elicited by creation of aorto-caval fistula (ACF) in male and female Lewis rats. * P<0.05 compared with intact healthy native heart. ^#^ P<0.05 compared with intact ACF native heart. ^@^ P<0.05 compared with ACF transplanted heart.

Figure 5A shows that implantation of the expander 14 days after HT_x_ significantly attenuated the decreases in whole HW in ACF male rats (−31±1 vs. −62±2 %, P<0.05) as well as in ACF female rats (−31±1 vs. −55±1 %, P<0.05). The implantation of the expander also significantly attenuated the decreases in LVW as compared with those without expander implantation, this was so in ACF male rats as well as in ACF female rats (Fig. 5B). In contrast, implantation of the expander did not have any significant effect on HT_x_-induced RVW decreases in ACF male rats or in ACF female rats (Fig. 6C).

Figure 6 summarizes the parameters of myocyte size, specifically the cardiomyocyte width (CW), cardiomyocyte length (CL), and the ratio of CL to CW of native hearts from healthy (i.e. without creation of ACF) and ACF rats and transplanted hearts from ACF rats without expander implantation and with implanted expander, measured 14 days after HT_x_ in male and female rats.

As shown in Figure 6A, CW in native hearts of ACF rats was significantly higher than CW in native hearts of healthy rats, this was so in males (26.26±0.21 vs. 24.38±0.14 μm, P<0.05) and in females (25.55±0.12 vs. 23.39±0.18 μm, P<0.05). Mechanical unloading by HT_x_ in hearts from ACF rats resulted after 14 days in significant decreases in CW, which were markedly lower than in native hearts of ACF in male (20.98±0.24 vs. 26.26±0.21 μm, P<0.05) as well as female rats (20.41±0.14 vs. 25.55±0.12 μm, P<0.05). The implantation of the expander significantly attenuated decreases in CW elicited by mechanical unloading of the heart after HT_x_ as compared with those without expander implantation; this was so in the hearts from ACF, similarly in male (22.46±0.17 vs. 20.98±0.24 μm, P<0.05) and female rats (21.79±0.14 vs. 20.41±0.14 μm, P<0.05).

As shown in Figure 6B, in native hearts of ACF rats CL was significantly greater than CL in native hearts of healthy rats, similarly in male (161.3±0.54 vs. 144.9±0.22 μm, P<0.05) and in female rats (154.4±0.97 vs. 141.6±0.27 μm, P<0.05). Mechanical unloading by HT_x_ with or without expander implantation, did not alter CL in hearts from ACF rats, both in male and female rats.

As shown in Figure 6C, the corresponding increases in CW and CL in native hearts of ACF rats, both males and females, did not change the ratio of CL to CW in those animals, unlike the change observed in native hearts of healthy rats. In contrast, association of augmented decreases in CW and maintained CL in mechanically unloaded hearts from ACF rats resulted in marked increases in the CL to CW ratio as compared with values observed in native hearts of healthy rats, both males (7.71±0.11 vs. 5.99±0.04 μm, P<0.05) and females (64±0.05 vs. 6.07±0.05 μm, P<0.05). Implantation of the expander significantly attenuated increases in the CL to CW ratio elicited by mechanical unloading of the heart after HT_x_ as compared with the ratio measured in rats with or without expander implantation, this was so both in the hearts from ACF male and female rats.

## Discussion

***The first important set of findings*** of the present study relates to our observation of the absence of important differences in the process of cardiac atrophy after HT_x_ in the failing hearts between male and female rats, similarly in intact or gonadectomized animals. These data strongly suggest that in the failing hearts there are no important sex-linked differences in the course of unloading-induced cardiac atrophy. In addition, since gonadectomy did not exhibit any effects on the course of cardiac atrophy after HT_x_ in the failing hearts, male or female, the data suggests that in failing hearts there are no important effects of sex hormones on this process, and the differences are rather due to the inherent properties of the failing (donor’s) hearts. We are aware that this conclusion is valid only for so called “activational” actions of sex hormones (i.e. actions that are related to the presence of sex hormones in the circulation) and not to the actions known as “organizational” (actions that persist long after sex hormones have been removed from the circulation) [67,68]. The notion that in the process of unloading-induced cardiac atrophy the inherent properties of the transplanted heart (rather than hormonal conditions of the recipient) play critical role has been confirmed by our recent studies when female heart was transplanted into a male recipient and vice versa: it was shown that the course of unloading-induced cardiac atrophy was not altered when compared with the situation when donor’s hearts were transplanted into the recipient of the same sex [53].

The finding of the absence of sex-linked differences in the development of post-HT_x_ cardiac atrophy in the failing hearts is surprising and in striking contrast to our recent discovery that there are important sex differences in the process of unloading-induced cardiac atrophy in the healthy hearts [52,53]. We cannot offer any clear explanation of this discrepancy. We have taken into consideration the possibility that our present findings might be (or not) influenced by different initial values. In accordance with Wilder’s law of initial value in biological sciences [69], the relative (percent) change from the original level depends on the initial level of the examined function: with different initial values it is unclear if such differences in response to experimental manipulation are a real effect or a statistical artifact [69]. However, we showed that when HW, LVW and RVW values were normalized to the length of tibia (correct way to express the basal values of cardiac mass, standardly employed in our laboratory [52–54,60–64,71]), there were no significant differences between the weights of the native hearts, from intact or castrated male and female ACF animals at the initial time points (9 or 10 weeks after ACF creation) (Fig. 3). These findings argue against the possibility that the differences in basal values of cardiac mass could be responsible for the absence of sex-linked differences in the development of unloading-induced cardiac atrophy.

Furthermore, as in our previous studies [40,54], we noticed here that the degree of cardiac atrophy in the failing hearts (observed in the present and our recent studies [52,53] in the healthy – non-failing – hearts) is distinctly higher in the failing hearts. Notably, 7 days’ unloading by HT_x_ in failing hearts caused 48±2 % decrease in whole HW in ACF male rats (average from intact and gonadectomized animals, present study). In our recent study [52] in the healthy hearts the unloading resulted in 28±1 % decrease in whole HW (again, pooled values from intact male and gonadectomized male animals). These findings are of special importance at least for two reasons.

First, irrespective of the absence of sex-linked differences in the process of unloading-induced cardiac atrophy in the failing hearts, the findings of augmented cardiac atrophy after HT_x_ in the failing hearts support our notion that cardiac atrophy might represent critically important detrimental effect of the prolonged LVAD-induced mechanical unloading, which might offset the beneficial influence of the process of “LV reverse remodeling”. Therefore, functional LV improvement is not translated into the clinical outcomes. This notion is equally valid for both sexes.

Second, our present findings further support the notion and our firm belief that potential anti-atrophic measures aimed at attenuation of unloading-induced cardiac atrophy should be tested in the failing hearts, even though such experiments are technically more difficult than the studies using healthy hearts. Unfortunately, the majority of relevant experimental studies was performed in healthy hearts even though this limitation was repeatedly admitted [24,28,29, 35–39,41,43]. To our knowledge only our group has been using the failing hearts in studies evaluating the process of unloading-induced cardiac atrophy using the model of heterotopic HT_x_ [40,41,54].

We cannot explain the reason/s for the enhanced unloading-induced cardiac atrophy in the failing hearts as compared with the healthy hearts, however, at least two possibilities should be considered.

The first is that in the case of the failing hearts the more pronounced unloading-induced cardiac atrophy (versus healthy hearts) is the consequence of higher initial baseline levels for cardiac mass in ACF animals. In other words, in accordance with what was originally suggested by Wilder for any similar circumstances [69], augmented decreases in cardiac mass might be the consequence of the fact that basal values of HW for ACF animals were about twice higher than those observed in healthy animals (no ACF) (see the data in our recent studies – ref. #^52^ and 53) . Therefore HT_x_-induced unloading elicited higher percent decreases in the failing hearts as compared with healthy hearts.

The second point to be considered is that in our study the hearts from the animals with advanced HF (known to be accompanied by marked activation of neurohormonal systems in the circulation [70,71]) were abruptly placed in normal neurohormonal environment of healthy animals. This situation could have some modulatory influence on the course of unloading-induced cardiac atrophy, which presents another limitation of our experimental techniques and all our studies employing HT_x_ technique. As discussed above, ACF animals examined 9 and 10 weeks after creation of ACF are in the advanced stage of HF and cannot serve as recipients, simply because they would not survive the HT_x_ procedure (the perioperative and acute postoperatively mortality would be extremely high). Nevertheless, considering the unsolved problems with the current findings, performing such technically extremely demanding studies seems to be critically important.

***The second important set of findings*** relates to our observation that enhancement of isovolumic loading induced by implantation of the spring expander into the LV substantially attenuated the process of cardiac atrophy after HT_x_ in the failing hearts, and the degree of attenuation was almost identical in male and female rats. This was documented by higher whole HW and LVW of the transplanted hearts provided with the expander than in the hearts without it (summarized in Tables 1 and 2). This was also evident from smaller decreases in whole HW and LVW of the transplanted hearts with implanted expander compared with the decreases in the hearts without the expander (calculated as percent decreases of the transplanted hearts in relation to the control native heart from ACF animals, Fig. 5).

All these findings at the whole organ level were also corroborated at the cardiomyocyte level. We found that there were no significant differences in the CW, CL and in the CL to CW ratio in the native healthy hearts (i.e. non-failing, without ACF creation) between male and female rats. Creation of ACF caused proportional increases in CW and CL and unchanged CL to CW ratio, again similarly in male and female rats. These findings confirm again the absence of sex-linked differences between the native healthy hearts and the native failing hearts at the cardiomyocyte level. In addition, the data for ACF animals corroborate again the trace presence of eccentric cardiac hypertrophy. Moreover, our data show that HT_x_ (per se) caused marked decreases in cardiomyocyte width (CW) and no change in cardiomyocyte length (CL), resulting in an increase in the CL to CW ratio; again the change was comparable in male and female rats. This was an evident sign of HT_x_-induced cardiac atrophy at the cardiomyocyte level. However, in our opinion the most important finding here was that implantation of the spring expander attenuates decreases in CW after HT_x_ without altering CL, similarly in male and female rats. This further corroborates the general conclusion that increasing isovolumic loading attenuates unloading-induced cardiac atrophy.

In this context, it should be remembered that the concept to increase isovolumic loading as an anti-atrophic measure was inspired by a pioneering study by Klein and co-workers [72] who found that insertion and subsequent inflation of a latex balloon into the LV increases isovolumic loading and markedly attenuates the development of cardiac atrophy after HT_x_ in the healthy hearts. Unfortunately, this approach is not applicable in the clinic, because it would cause obstruction of the LV and failure of cardiac output. Therefore, these findings were long disregarded. However, the Klein’s study provided a crucial inspiration for us and for development of the present spring expander, which provides a sufficient isovolumic loading without impairment of ejection function of the LV and also without damage of the LV structures, also of the aortic valve [41,54]. Our hope was that so obtained enhanced isovolumic loading would attenuate unloading-induced cardiac atrophy. After considering our previous [54] and present findings and the common knowledge that cardiac work is one of the major determinants of the size and growth of the heart [73–75], we propose that enhancing cardiac work due to increased isovolumic loading obtained by implantation of the expander is a reasonable approach to attenuate the unloading-induced cardiac atrophy in the failing hearts; this should be effective in both sexes.

Our present findings are of special interest because the available data from the clinical database still appear to indicate an important sex-related disparity of the results of LVAD application for the treatment of advanced HF [48–51,76–78]. Our present data clearly show that there are no sex-linked differences in the course of unloading-induced cardiac atrophy and also in the response to the spring expander implantation into the LV after HT_x_. Therefore, we believe that sex differences in the failing hearts, i.e. in the situation of the advanced HF, should not be considered as an important factor in the search for treatment measures against unloading-induced cardiac atrophy.

## Conclusions

On the whole, our present data clearly show that there are no sex-related differences in the development of unloading-induced cardiac atrophy in the failing hearts. In addition, we found that induction of isovolumic loading by implantation of the three-branch spring expander into the LV attenuates the process of unloading-induced cardiac atrophy in the failing hearts; this process and the degree of attenuation is similar in both sexes. Nevertheless, it is important to recognize that future studies are urgently needed to elucidate the reasons of the absence of sex-linked differences in the process of unloading-induced cardiac atrophy.

## Figures and Tables

**Table 1:** The weight of transplanted (i.e. donor) heart and the native heart and of the individual heart structural components after heterotopic heart transplantation (HT_x_). Native heart values served as basal values (100 %) for evaluation of the process of cardiac atrophy in animals after HT_x_.

	Parameter							
	BW (g)	TK (mm)	HW (mg) (native)	HW (mg) (HT_x_)	LVW (mg) (native)	LVW (mg) (HT_x_)	RVW (mg) (native)	RVW (mg) (HT_x_)
*Group of males*								
*Intact male recipient + HT* *_x_* * of failing male donor’s heart (7 days after HT* *_x_* *)*	401 ± 10	40.8 ± 0.19		1055 ± 33		642 ± 19		202 ± 12
*Intact ACF male Lewis rats 9 weeks after creation of ACF*	397 ± 8	39.4 ± 0.25	2072 ± 103		1208 ± 51		466 ± 34	
*Intact male recipient + HT* *_x_* * of failing male donor’s heart (14 days after HT* *_x_* *)*	396 ± 9	40.1 ± 0.20		815 ± 37		498 ± 17		153 ± 8
*Intact male recipient + HT* *_x_* * of failing male donor’s heart+ implantation of expander (14 days after HT* *_x_* *)*	399 ± 9	40.2 ± 0.21		1432 ± 48*		953 ± 24*		161 ± 12
*Intact ACF male Lewis rats 10 weeks after creation of ACF*	391 ± 10	40.6 ± 0.22	2048 ± 92		1222 ± 49		451 ± 30	
*Castrated male recipient + HT* *_x_* * of failing male donor’s heart (7 days after HT* *_x_* *)*	379 ± 12	39.9 ± 0.20		1040 ± 57		654 ± 35		211 ± 13
*Castrated ACF male Lewis rats 9 weeks after creation of ACF*	368 ± 12	39.6 ± 0.21	2016 ± 93		1177 ± 55		471 ± 27	
*Castrated male recipient + HT* *_x_* * of failing male donor’s heart (14 days after HT* *_x_* *)*	384 ± 14	40.2 ± 0.22		807 ± 29		474 ± 22		167 ± 11
*Castrated ACF male Lewis rats 10 weeks after creation of ACF*	362 ± 15	40.1 ± 0.18	1999 ± 67		1184 ± 58		444 ± 25	

Values are means ± SEM. BW, body weight; TL, tibia length; HT_x_, heterotopic heart transplantation; HW, heart weight; LVW, left ventricle weight; RVW, right ventricle weight.

* P<0.05 compared with values from transplanted heart either intact or castrated at the same time point (i.e. 14 days after HT_x_).

**Table 2:** The weight of transplanted (i.e. donor) heart and the native heart, and of the individual heart structural components after heterotopic heart transplantation (HT_x_). Native heart values served as basal values (100 %) for evaluation of the process of cardiac atrophy in animals after HT_x_.

	Parameter							
	BW (g)	TK (mm)	HW (mg) (native)	HW (mg) (HT_x_)	LVW (mg) (native)	LVW (mg) (HT_x_)	RVW (mg) (native)	RVW (mg) (HT_x_)
*Group of females*								
*Intact female recipient + HT* *_x_* * of failing female donor’s heart (7 days after HT* *_x_* *)*	225 ± 6	35.5 ± 0.21		912 ± 38		512 ± 27		213 ± 8
*Intact ACF female Lewis rats 9 weeks after creation of ACF*	235 ± 7	35.9 ± 0.18	1565 ± 70		959 ± 37		352 ± 26	
*Intact female recipient + HT* *_x_* * of failing female donor’s heart (14 days after HT* *_x_* *)*	245 ± 7	36.1 ± 0.19		847 ± 25		501 ± 13		177 ± 16
*Intact female recipient + HT* *_x_* * of failing female donor’s heart+ implantation of expander (14 days after HT* *_x_* *)*	241 ± 8	35.8 ± 0.17		1093 ± 26*		763 ± 26*		172 ± 21
*Intact ACF female Lewis rats 10 weeks after creation of ACF*	245 ± 6	35.6 ± 0.22	1584 ± 65		966 ± 21		355 ± 25	
*Castrated female recipient + HT* *_x_* * of failing female donor’s heart (7 days after HT* *_x_* *)*	247 ± 7	36.1 ± 0.21		943 ± 39		614 ± 24		199 ± 7
*Castrated ACF female Lewis rats 9 weeks after creation of ACF*	274 ± 12	35.9 ± 0.22	1572 ± 59		942 ± 56		339 ± 25	
*Castrated female recipient + HT* *_x_* * of failing female donor’s heart (14 days after HT* *_x_* *)*	270 ± 9	36.1 ± 0.18		825 ± 42		524 ± 13		178 ± 9
*Castrated ACF female Lewis rats 10 weeks after creation of ACF*	282 ± 13	36.5 ± 0.23	1569 ± 63		970 ± 31		359 ± 27	

Values are means ± SEM. BW, body weight; TL, tibia length; HT_x_, heterotopic heart transplantation; HW, heart weight; LVW, left ventricle weight; RVW, right ventricle weight.

* P<0.05 compared with values from transplanted heart either intact or castrated at the same time point (i.e. 14 days after HT_x_).
